# Dysregulation of dopamine neurotransmission in drug addicts: implications for criminal behavior and corrective interventions

**DOI:** 10.3389/fpsyt.2024.1434083

**Published:** 2024-11-25

**Authors:** Chao Gu, Yu-chang Geng, Li-na Zhu

**Affiliations:** ^1^ Law School, Yangzhou University, Yangzhou, China; ^2^ College of Physical Education, Yangzhou University, Yangzhou, China

**Keywords:** corrective measures, criminal behavior, dopamine, physical, drug addiction

## Abstract

Drug addiction often correlates with criminal behavior. When investigating criminal behavior among individuals grappling with drug addiction, it becomes crucial to scrutinize the influence of dopamine. Substances such as heroin, morphine, methamphetamine and other drugs can cause abnormal dopamine secretion when people are addicted to them, which promotes changes in the brain’s reward circuit and emotional balance, thereby increasing susceptibility to criminal behavior. The pivotal role of dopamine within the reward pathway and its regulatory function in emotional processes exert profound influence on behavior following drug simulation. These influences are primarily manifested by three distinct attributes: a singular criminal motive and objective, lack of moral sense, and impulsive decision-making processes. Drawing upon the distinctive dopaminergic dynamics inherent in individuals afflicted by drug addiction, this study advocates for targeted corrective interventions. The preventive paradigm encompasses the cultivation of supportive community environments, the establishment of comprehensive databases, and providing legal education and protection, among other initiatives. In terms of treatment, along with judicial sanctions and protections, exercise regimens and psychotherapeutic interventions are advocated. The corrective endeavor necessitates a synergistic integration of community-based and legalistic frameworks. The objective is to furnish guiding principles for tackling criminal behavior precipitated by aberrant dopamine secretion, underpinned by a scientifically informed approach.

## 1 Introduction

Drug addiction represents a significant global challenge in public health, transcending mere health concerns to impact societal dynamics and criminal activity (1). In relevant research, for example, drugs such as cannabis are closely associated with psychosis (2), which can lead to criminal behavior (3). Looking deeper into the reasons, it is found that a neurotransmitter “dopamine” is indispensable. The neurotransmitter dopamine, pivotal in mediating motivational processes, emotional responses, and cognitive functions include learning and memory, assuming a central role in this context (4). However, within the context of drug addiction, dysregulated dopamine activity precipitates the onset and perpetuation of criminal behaviors. Therefore, a comprehensive examination of the pivotal role of dopamine in driving and sustaining such behaviors within the context of drug addiction becomes imperative. This necessitates a meticulous assessment of the distinctive attributes and repercussions of dopamine function in engendering criminal conduct. Also, a comparative analysis between the criminal behaviors exhibited by individuals struggling with drug addiction and those observed in the general populace is essential to grasp the intricate nuances of criminal behavior within the framework of drug addiction. Such a comparative approach promises a more nuanced understanding of the multifaceted nature of criminal behavior amidst drug addiction. By furnishing valuable insights into this intricate interplay, this study stands to enrich the knowledge base of researchers operating within the domains of public health and law. Also, it provides insights for policymakers, helping them formulate efficacious strategies for both preventing and reducing the harmful impact of drug addiction-associated criminal behaviors.

## 2 Methods

### 2.1 Literature search

A systematic search was conducted across multiple databases, including PubMed, Web of Science, and Scopus. The search terms included “dopamine”, “drug addiction”, “criminal behavior”, and “corrective interventions”. The search was limited to English-language articles published within the last 30 years.

### 2.2 Study selection

Articles were selected based on their relevance to the research question. Inclusion criteria were:

(1) studies that investigated the relationship between dopamine and drug addiction; (2) studies that explored the impact of drug addiction on criminal behavior; (3) studies that proposed or evaluated corrective interventions for drug addicts’ criminal behavior. Exclusion criteria were: (1) non-original research (e.g., review, metanalysis, commentary, editorial, letter to the editor without data available, and book chapter); (2) non-full-text articles (e.g., meeting abstract); (3) language other than English; (4) animal/*in vitro* studies; (5) articles not related to custodial setting; (6) articles not related to selected substances.

### 2.3 Data extraction

For each selected article, the following data were extracted: (1) study design (e.g., experimental, observational, review); (2) sample size and characteristics (e.g., age, gender, drug of choice); (3) methods used to measure dopamine (e.g., neuroimaging, biochemical assays); (4) methods used to assess criminal behavior (e.g., self-report, criminal records); (5) details of the proposed or evaluated corrective interventions. Data Synthesis: The extracted data were synthesized and analyzed qualitatively. The relationships between dopamine, drug addiction, and criminal behavior were explored, and the effectiveness of different corrective interventions was evaluated.

### 2.4 Results of the included literature

**Figure 1:**
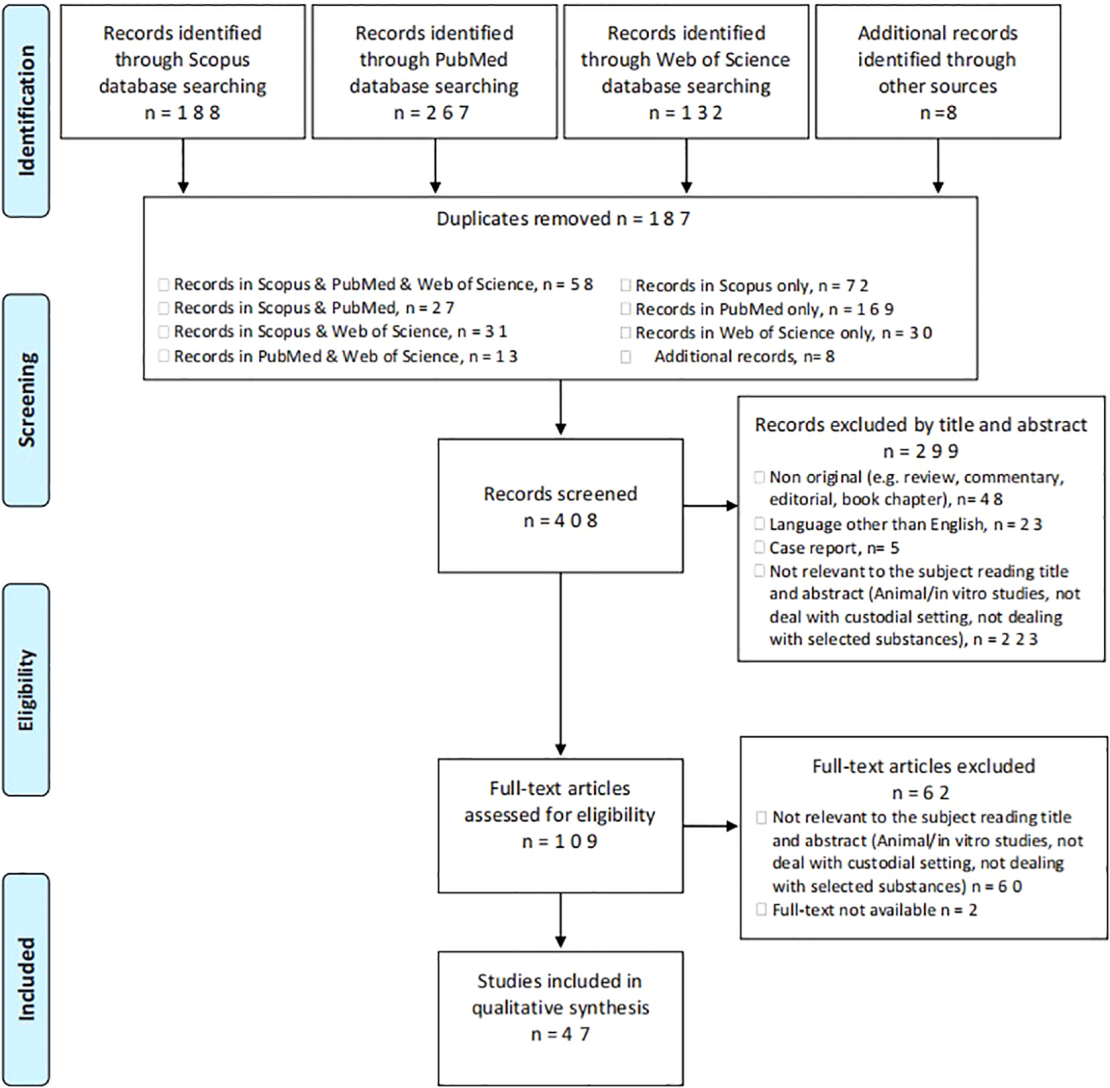
Prisma flow diagram of the methodology of the systematic literature review.

From a total of 595 articles (PubMed = 267; Scopus = 188; WoS = 132; other sources = 8), after deduplication (n = 187), 408 records were screened. Among the articles screened, 223 were considered not relevant to the subject after reading the title and abstract, 23 were not written in English, 48 were non-original articles and 5 were case-reports. Of the 109 full-text articles assessed for eligibility, 62 did not match the inclusion criteria for our review; finally, 47 articles were included in the systematic review (**Figure 1**) (5).

## 3 The impact of drug addiction on dopamine secretion

### 3.1 Characteristics of dopamine in the reward mechanism

Dopamine serves as a key neurotransmitter within the intricate neural circuitry of the brain, exerting profound influence over an array of psychological and physiological processes (6). Its role within the reward pathway of the brain is of particular significance, where it acts as a potent motivator, propelling individuals towards the pursuit of positive behavior. Upon encountering such stimuli or engaging in positive behaviors, the dopaminergic system orchestrates the release of dopamine, facilitating its transmission to various brain regions. This transmission allows individuals to experience sensations of pleasure and satisfaction, thereby imprinting these experiences within the cognitive framework of the individual and instigating a cyclical pattern of seeking similar gratification, a phenomenon colloquially known as ‘reinforcement learning’ within biological contexts (7). However, the use of drugs disrupts this finely tuned dopaminergic equilibrium, precipitating abnormal dopamine release within the brain.

In the context of drug addiction, individuals are subjected to intense euphoria due to the excessive dopaminergic surge induced by drug ingestion. Consequently, through reinforcement learning mechanisms, their responsiveness to drug-related cues becomes intensified, fostering an insatiable craving for drugs. This insatiable craving, in turn, assumes the role of a primary motivator, propelling individuals towards engaging in criminal behaviors, ultimately resulting in severe consequences (8).

Numerous studies underscore the role of dopamine as a “predictive” neurotransmitter, signifying its activation during the anticipation of rewards. This activation engenders pleasurable sensations, propelling individuals towards seeking the anticipated reward, a phenomenon integral to motivating participation in work and learning endeavors. However, in the context of individuals struggling with drug addiction, the involvement of dopamine in anticipatory processes assumes heightened significance, leading to a more intense dopaminergic response upon the anticipation of drugs, eclipsing responses evoked by conventional natural rewards (9).

Consequently, the brains of individuals afflicted by drug addiction become hypersensitive to drug-associated stimuli, encompassing individuals providing drugs, drug-related environments, and drug-related behaviors, all of which elicit robust neural activation. Upon exposure to these drug-associated cues, a confluence of reinforcement learning mechanisms and heightened anticipatory processes triggers a surge in dopamine release within the brain, culminating in intense anticipation and craving for drugs (10).

Within the framework of drug addiction, the brain undergoes a process of tolerance development owing to the prolonged exposure to excessive dopamine stimulation. This phenomenon necessitates individuals to escalate drug doses to attain the same level of pleasure, thereby accentuating the importance of tolerance within the addiction trajectory. Prolonged drug consumption precipitates enduring changes in the dopamine signaling pathways of the brain, culminating in a state termed ‘anhedonia,’ wherein addicts find themselves incapable of deriving pleasure from conventional rewards after quitting drugs. Consequently, this phenomenon explains the high risk of addicts to relapse, even subsequent to successful detoxification efforts. The profound influence of drugs rewires the reward system in their brains, compelling individuals to prioritize drug-related rewards over those inherent in healthy natural stimuli (11).

### 3.2 The role of dopamine in emotional regulation

Dopamine, a key neurotransmitter intricately involved in the regulation of emotions, exerts profound influence over the emotional well-being of an individual. The experience of happiness and excitement prompts specific brain regions to unleash heightened dopamine levels, thereby instigating a cascade of positive emotions (12). However, the landscape of emotion is significantly changed in drug addiction, as profound perturbations within the dopamine system precipitate consequential shifts in the emotional state of an individual. Substances such as heroin, cocaine, and methamphetamine elicit rapid surges in dopamine levels within the brain, surpassing its natural physiological thresholds. This leads to transient episodes of intense pleasure, swiftly succeeded by precipitous declines in dopamine levels, leading to negative emotions (13).

Sustaining stable dopamine levels is essential for fostering emotional equilibrium and facilitating the smooth execution of occupational and daily activities. However, prolonged use of drugs inflicts substantial damage upon the dopamine systems of individuals ensnared by addiction, resulting in serious problems in emotional regulation. Post-drug administration, dopamine levels undergo abnormal fluctuations, leading towards extremes of excess or deficiency, thereby instigating long lasting manifestations of depression, anxiety, emotional volatility, and irritability (14). Also, the acclimatization of the brain to heightened dopamine stimulation engenders transient dysfunction within the dopamine system when the individuals stop using drugs, leading to the emergence of withdrawal symptoms (15).

These symptoms, characterized by an overwhelming urge for drugs, frequently prompt individuals dealing with addiction to flout legal, social, and ethical norms, underscoring a conspicuous absence of moral discernment. This lack of moral sensibility assumes a pivotal role in propelling drug-dependent individuals towards engagement in criminal activities. The underlying cause of all these problems is the disruption wrought upon the dopamine system by drugs, which impairs the normative regulation of emotional states, thereby exacerbating drug dependency, compromising decision-making faculties, and heightening susceptibility to relapse among individuals trapped by addiction.

## 4 The impact of abnormal dopamine secretion on criminal behavior

### 4.1 Relative singular criminal motive and goal

In the field of criminology, criminal behavior refers to actions that violate criminal law and is characterized by their danger and destructiveness, thereby inflicting substantial harm upon societal fabric.

General criminal conduct encompasses various types of offenses, each driven by different motives and objectives, shaped by an interplay of economic, psychological, and socio-environmental factors. Economic factors, such as unemployment, financial strain, or existential pressures, may propel individuals towards criminality as a means of enhancing their economic circumstances, be it through acts of theft, robbery, fraud, or similar transgression (16). Conversely, individual psychological factors, encompassing mental illness, emotional disturbances, or personality disorders, can impel certain individuals towards non-conformity with societal norms, stemming from conditions like antisocial personality disorder, psychological trauma, or impulse control disorders. These individuals may resort to criminal pursuits to satiate their craving for stimulation and gratify their distorted psychological needs (17). Also, socio-environmental factors, such as group dynamics, societal pressures, and aspirations for social status, can also contribute to criminal behavior. Within impoverished and crime-ridden communities, engagement in illicit activities may be construed as a means of attaining social recognition or gaining social status, thereby fortifying or perpetuating a way to gain social standing within the community fabric (18).

Furthermore, individuals grappling with drug addiction typically demonstrate a singular focus in their motives and goals. Admittedly, psychological and socioeconomic factors affect the criminal behavior of drug addicts, but their criminal conduct is predominantly propelled by an overwhelming craving for and reliance upon drugs, collectively known as ‘addiction-driven crime.’ Their primary objective revolves around securing additional drugs or acquiring the resources necessary for their procurement (19). Unlike general criminal behavior, which often manifests as a complex interplay of diverse social, economic, and psychological factors, the criminal actions of individuals trapped by addiction are predominantly steered by the urgency to satiate their drug dependency, with minimal influence from other contextual elements.

Individuals afflicted by drug addiction frequently struggle with enduring anxiety and depression, prompting them to seek solace through drug consumption. Despite providing transient relief, drugs invariably precipitate exacerbated withdrawal symptoms, perpetuating a destructive cycle wherein individuals escalate their substance intake to more drugs to alleviate these adverse effects. Consequently, this cycle engenders a deepening of addiction, culminating in the fortification of illegal and criminal behaviors. Addiction-driven criminal conduct is hallmarked by heightened impulsivity and a conspicuous disregard for repercussions, as drugs exert profound changes upon the dopamine system within the brains of afflicted individuals, hindering their capacity for reasoned decision-making and emotional regulation.

### 4.2 Lack of moral sense

To thoroughly understand and compare the nuances in moral discernment between general criminal behavior and criminal conduct observed in individuals struggling with drug addiction under the influence of dopamine, a multifaceted inquiry is necessary, drawing upon diverse perspectives and disciplinary frameworks.

Criminal behavior frequently correlates with deficits in moral reasoning (20). By using sociological lens, offenses such as theft, robbery, and violence are construed as transgressions against societal norms and moral precepts, indicative of a callous disregard for the rights of others and the sanctity of their lives and possessions. Various intricate factors, such as economic exigencies, psychological afflictions, and socio-environmental influences, converge to engender a lack of adherence to moral dictates and social conventions, thereby culminating in a blunted moral sensibility. In an attempt to reduce conflicts and psychological stressors, individuals may rationalize their actions under the guise of self-defense, thereby seeking moral justification for their conduct (21).

In individuals dealing with drug addiction, the dopamine system of the brain undergoes profound adaptation to the effects of drugs, precipitating a decline in their moral acumen and societal obligations. In normal circumstances, human behavior is governed by the reward circuitry of the brain, which modulates the intensity and frequency of actions based on their outcomes. When behavior aligns with societal norms and moral standards, the reward system furnishes positive reinforcement, encouraging individuals to perpetuate positive behavior However, individuals with drug addiction have already experienced pronounced adaptation within the reward circuitry of their brain to the allure of drug-induced rewards. Consequently, they tend to disregard or minimize the adverse consequences of behaviors contravening societal norms and moral precepts, instead prioritizing the gratification of their drug cravings above all else (22).

Also, the lack of moral sense involves intricate underlying physiological and psychological factors, compounded by substantial changes within the dopamine system of individuals afflicted by drug addiction (23). This engenders robust cravings and dependence on drugs, potentially reducing their moral sensibilities and social obligations. Consequently, individuals struggling with drug addiction may prioritize the satisfaction of their intense drug cravings over contemplating the adverse ramifications of their actions on their families and broader society. As they resort to theft, robbery, or acts of violence to procure drugs, their cognitive faculties may disregard or diminish the moral assessments and self-reproach associated with such behaviors.

### 4.3 Impulsivity in decision-making

There are notable differences that emerge in the decision-making processes underpinned by dopamine between general criminal behavior and the criminal conduct manifested by individuals who are addicted to drugs.

General criminal behavior is characterized by a multifaceted cognitive calculus, wherein individuals deliberate upon a series of factors. They meticulously assess the potential benefits associated with engaging in criminal acts, ranging from economic gains to enhancement in social stature, or the fulfillment of emotional and psychological exigencies. Simultaneously, they weigh the prospective negative repercussions of their actions, encompassing legal ramifications, societal censure, and damage of personal reputation. For instance, in the case of a theft, individuals may contemplate the economic windfall juxtaposed with the legal and social hazards that may result from their proposed actions. Their decision-making paradigm is frequently influenced by personal morals, values, and their perception of societal equity (24).

Furthermore, individuals struggling with drug addiction manifest distinct decision-making paradigms under the influence of dopamine. Prolonged substance abuse leads to profound changes within the brain’s reward circuitry, thereby exerting a transformative impact on their decision-making processes. They often exhibit a pronounced proclivity towards prioritizing the immediate gratification derived from drug consumption, irrespective of the potential health ramifications, interpersonal discord, or legal entanglements entailed by criminal behavior. Similarly, their decision-making framework becomes significantly influenced with impulsivity, wherein the allure of immediate drug-induced euphoria eclipses considerations of long-term consequences. Also, the decision-making trajectory of individuals who are addicted to drugs is further compounded by intense drug cravings. These cravings generate potent anticipations of reward within their neural substrates, thereby predisposing them towards actions aimed at obtaining and using drugs, while concurrently relegating different choices that may confer long-term benefits to the periphery. This elucidates why many individuals struggling with drug addiction persist in engaging in criminal activities, notwithstanding the absence of immediate financial exigencies (25).

The emotional fluctuations experienced by individuals with drug addiction exert a profound influence on their decision-making processes, exacerbated by the disruptions of the dopamine system. During periods of emotional distress, individuals may resort to drug consumption as a means of quickly elevating dopamine levels and alleviating feelings of despondency. However, in this state, their decision-making framework tends to prioritize immediate relief over contemplation of long-term consequences, leading to high-risk behaviors (26). In fact, when individuals find themselves in a heightened emotional state, they may manifest a proclivity towards overly optimistic biases in their decision-making. This predisposes them towards disregarding potential risks and challenges, instead fixating excessively on immediate rewards. Such decision-making biases serve to exacerbate addictive behaviors and increase the likelihood of engaging in dangerous activities, including criminal conduct (27).

## 5 Advocating for correction measures for drug addicts’ criminal behavior

### 5.1 Prevention of criminal behavior by drug addicts

Preventing drug addiction constitutes a pivotal measure in reducing the likelihood of initial drug experimentation and subsequent dependence and criminal activities.

Given the critical role of dopamine in stress management and simulation, prevention strategies should focus on bolstering the resilience of individuals facing addiction and facilitating the cultivation of healthier lifestyles, which will enhance their ability to cope with stress (28). Communities can provide mental health education initiatives that explain the impact of stress on the dopamine system and physiological well-being. These programs empower individuals struggling with addiction to recognize the viability of using constructive methods to regulate emotions, rather than resorting to drugs as an escape mechanism.

Interactive teaching approaches such as role-playing or situational simulations can be incorporated into these initiatives, enabling participants to acquire and rehearse stress management and emotional regulation skills within real-life scenarios. By reducing the likelihood of initial drug exposure and promoting adaptive coping mechanisms, these programs can foster the development of healthier and more constructive approaches to stress management. Furthermore, high-risk groups, including individuals from fractured family backgrounds, those contending with mental health challenges, individuals struggling with trauma, and those with a history of substance dependencies, need more support and attention. Providing personalized psychological counseling and social services can help these vulnerable populations with the requisite tools to address underlying issues, fostering emotional regulation and behavioral modulation, thereby reducing the propensity towards drug use and engagement in criminal behaviors. Also, fortifying community-based educational endeavors concerning the dopamine system holds merit. These educational initiatives serve to enhance public awareness regarding the intricacies of dopamine system functioning, thereby facilitating early detection of dopamine imbalances and preventing the exacerbation of addiction-related symptoms.

The establishment of a comprehensive dopamine database emerges as a pivotal endeavor for consolidating relevant information relating to dopamine, encompassing early indicators of dopamine system imbalances and changes in dopamine levels throughout the trajectory of drug addiction. This repository serves as an invaluable resource for professionals, furnishing them with requisite insights to facilitate accurate diagnosis of drug addiction and effective prevention of both substance dependency and associated criminal behaviors.

Continual updates to the database are key to ensure alignment with the latest advancements stemming from technological and medical research. Also, the creation of an expansive database tailored to individuals with drug addiction holds immense promise. By collecting and analyzing diverse datasets relating to drug use—such as temporal, demographic, and socio-economic parameters, along with specific physiological and neurobiological sequelae—the database affords researchers a comprehensive framework to give insights into trends and patterns underpinning addiction trajectories. This culminates in a nuanced comprehension of the intricate dynamics surrounding drug addiction, thereby facilitating proactive intervention strategies to preempt the onset of criminal behaviors.

Leveraging big data analytics further increases the use of these databases, facilitating the assessment of extant prevention and treatment modalities. By comparing data pre- and post-implementation of intervention strategies, critical insights can be discovered, enabling the identification of areas that need to be refined as well as assessment of efficacy. This fosters targeted reforms with the goal at continual optimization of prevention and treatment paradigms, thereby efficaciously preventing the incidence of drug addiction and associated criminal conduct.

The educational aspect of the law assumes paramount significance within the ambit of prevention efforts. A comprehensive and multi-faceted legal education regimen can fortify the legal acumen of individuals with drug addiction, enabling them with a deeper understanding of the dire ramifications stemming from illicit drug use, while increasing their capacity to resist drug-related temptations (29). Community-based educational initiatives revolving around drug laws can be instrumental in engendering heightened legal consciousness among individuals with drug addiction. These educational endeavors may encompass courses elucidating legal responsibilities and delineating the profound impact of illicit drug use on familial and societal spheres. Community organizations can further enhance these efforts by regularly hosting lectures delivered by legal experts, who impart their professional insights and practical experiences, thereby affording individuals with addiction a more palpable grasp of legal intricacies.

Also, by leveraging the ubiquitous nature of the internet, governmental and non-governmental entities can harness online platforms to disseminate knowledge relating to drug laws. Various types of multimedia content, spanning articles, videos, and animations, can be disseminated to facilitate adaptable learning modes, catering to the diverse needs of individuals with drug addiction. Innovative interactive methodologies, such as online Q&A sessions and discussion forums, can be used to address queries and apprehensions, guiding individuals towards a more accurate assimilation and application of legal knowledge (30).

Criminal law plays a key role in deterring individuals with drug addiction from succumbing to criminal behavior. By defining specific charges and corresponding penalties for drug-related offenses, criminal law efficaciously underscores the gravity of drug-related transgressions and underscores the necessity of abiding by legal mandates. This not only raises awareness among individuals with addiction regarding the deleterious impact of drugs on the dopamine system but also serves as a potent deterrent. For instance, within the legal framework of China, the Criminal Law encompasses provisions relating to various drug-related offenses, ranging from ‘drug smuggling, trafficking, transportation, and manufacturing’ to ‘knowingly harboring drug users’ and ‘illegal possession of drugs’.

These provisions conspicuously stipulate that engagement in drug-related crimes will lead to severe legal repercussions. Such statutory provisions wield considerable efficacy in dissuading the public from involvement in drug addiction and associated criminal activities, thereby forestalling the perpetuation of the cycle of substance dependency. Also, criminal law introduces various prevention and control measures such as compulsory drug rehabilitation initiatives, with the objective of facilitating the restoration of normal brain function among drug addicts, decreasing reliance on drugs, and implementing behavioral regulation and guidance. Also, stringent legal sanctions serve to forestall more individuals from causing damage to their dopamine systems thanks to drug-induced neurochemical disturbances. In cases of egregious drug offenses, criminal law imposes severe penalties, such as the imposition of the death penalty, which serves as a potent admonition and deterrent to both individuals with addiction and prospective drug offenders. In summary, criminal law emerges as an indispensable instrument in the prevention of drug-related crimes and the facilitation of addiction recovery, achieved through the delineation of charges and penalties, implementation of preventive measures, and rigorous crackdowns on drug-related crimes.

### 5.2 Treatment for drug addicts’ criminal behavior

Treatment for drug addicts who have engaged in criminal behavior is critically important. Integral to this approach is the enhancement of physical and mental health, enhancement of social competencies, and implementation of preventive measures to reduce relapse. Informed by an appreciation of the impact of drug addiction on the dopamine system, these interventions aspire to significantly enhance the holistic well-being of drug addicts, thereby deterring them from involvement in criminal activities.

Exercise therapy emerges as a promising adjunctive treatment modality for individuals with drug addiction and involvement in criminal behavior. Research indicates that enhancing physical health through exercise can expedite the recovery process, with exercise exerting effects similar to drugs by enhancing dopamine concentration and receptor binding, thereby leading to heightened pleasure during drug withdrawal (31). Research underscores the efficacy of exercise in reducing drug use among individuals with a history of substance exposure, presenting a non-pharmacological alternative to foster recovery (32).

Peretti-Watel et al. conducted a comparative study between adult athletes and non-athletes, revealing lower rates of drug use, abuse, and dependence among the former group (33). Similarly, Correia et al. demonstrated that escalating physical activity levels over a four-week interval correlated with diminished drug use among participants (34). Also, Roessler et al. observed a reduction in drug use among participants subjected to exercise intervention, indicative of early-stage addiction control (35). From this perspective, exercise therapy emerges as a compelling alternative treatment avenue capable of naturally stimulating the reward system of the brain, thereby satisfying drug cravings with fewer adverse effects compared to pharmacological interventions (36).

Cognitive-behavioral therapy (CBT) can be used to facilitate the recovery of individuals with mental health concerns. By changing the thinking patterns and behavioral habits, drug addicts can be assisted in better managing everyday life stressors and controlling their cravings for drugs (37). Damage to the dopamine system in the brain, caused by drug abuse, can affect the emotions and decision-making abilities of addicts. This often results in prioritizing short-term rewards over long-term consequences, leading to severe outcomes. Through CBT, drug addicts can change their thinking patterns and behavioral habits, allowing them to perceive the allure of drugs more logically. Emotion and behavior regulation through CBT also contributes to reducing the risk of relapse (38).

The significance of law in society cannot be disregarded, considering its substantial role. The objective of treatment is not solely to help addicts in overcoming drug addiction and regulating their dopamine system, but also to incorporate legal measures and safeguard their rights, facilitating their reform and reintegration into society. The Chinese Criminal Law adheres to the principle of proportionality between crimes and punishments, ensuring that the punishment for drug users corresponds to the severity of their offenses. By appropriately penalizing drug users, the goal is to safeguard the dopamine system, reduce further harm caused by drugs, and effectively prevent and control drug addiction and criminal behavior.

The Chinese Criminal Law also expressly upholds the principle of equality before the law, ensuring fair treatment of drug addicts without discrimination based on their drug use identity. Implementation of this principle ensures the protection of the rights of drug addicts during legal proceedings, ensuring not only legal fairness but also creating an honest legal environment for drug addicts, facilitating fair trials and appropriate punishment within legal boundaries. When imposing penalties, it is important to consider the biological characteristics of drug addicts and their impact on their dopamine systems, with the goal of guiding them towards rehabilitation while addressing their criminal behavior. Also, the law can provide necessary support and protection for treatment processes such as medication, exercise therapy, and mental health recovery.

Along with criminal law, the functioning of other legal frameworks is crucial for comprehensive support. Firstly, the law plays a pivotal role in establishing and safeguarding the rights of addicts. The US law, with its comprehensive and well-developed provisions in this area, can serve as a valuable reference for China. Based on US law, addicts have entitlements to access medical and psychological counseling services, fair education and employment opportunities, and other essential social support (39). These provisions not only safeguard the basic rights of addicts but also encourage their active participation in treatment, thereby enhancing treatment effectiveness. Secondly, the law can promote treatment by implementing relevant regulations and penalties. In foreign jurisdictions such as the UK, laws exist that allow participation in treatment as a condition for suspended sentences for addicts (40). These approaches incentivize addicts to voluntarily seek treatment and prevent them from facing harsher consequences.

Finally, it is crucial to establish various legal policies and systems that aid in the successful reintegration of addicts into society. Based on US law, drug addicts have the opportunity to apply for the expungement of their criminal records after completing successful drug rehabilitation. This provision aims to facilitate their access to employment and housing, ultimately aiding in their reintegration into society and enabling them to lead normal lives. These examples can serve as valuable references for future legal developments in China (41).

### 5.3 Correction of drug addicts’ criminal behavior

The primary focus of the correctional process is to assist drug addicts in their reform and successful reintegration into society. Considering the influence of dopamine on criminal behavior, it is essential to utilize a combination of community correction and legal measures to reduce the likelihood of becoming drug offenders again and facilitate successful reintegration.

The primary objective of community correction is to support drug addicts in gradually transitioning to a normal life within a gentle, yet consistently monitored environment (42). This process encompasses various elements such as community services, community monitoring, and the establishment of a stable support system. Community services can contribute to enhancing the public perception of drug addicts and enable their active involvement in community activities. These services may involve tasks such as cleaning public spaces, participating in community events, or providing assistance to the community. By engaging in these activities, drug addicts not only enhance their relationships within the community but also cultivate a stronger sense of self-worth and social responsibility (43).

**Figure 2:**
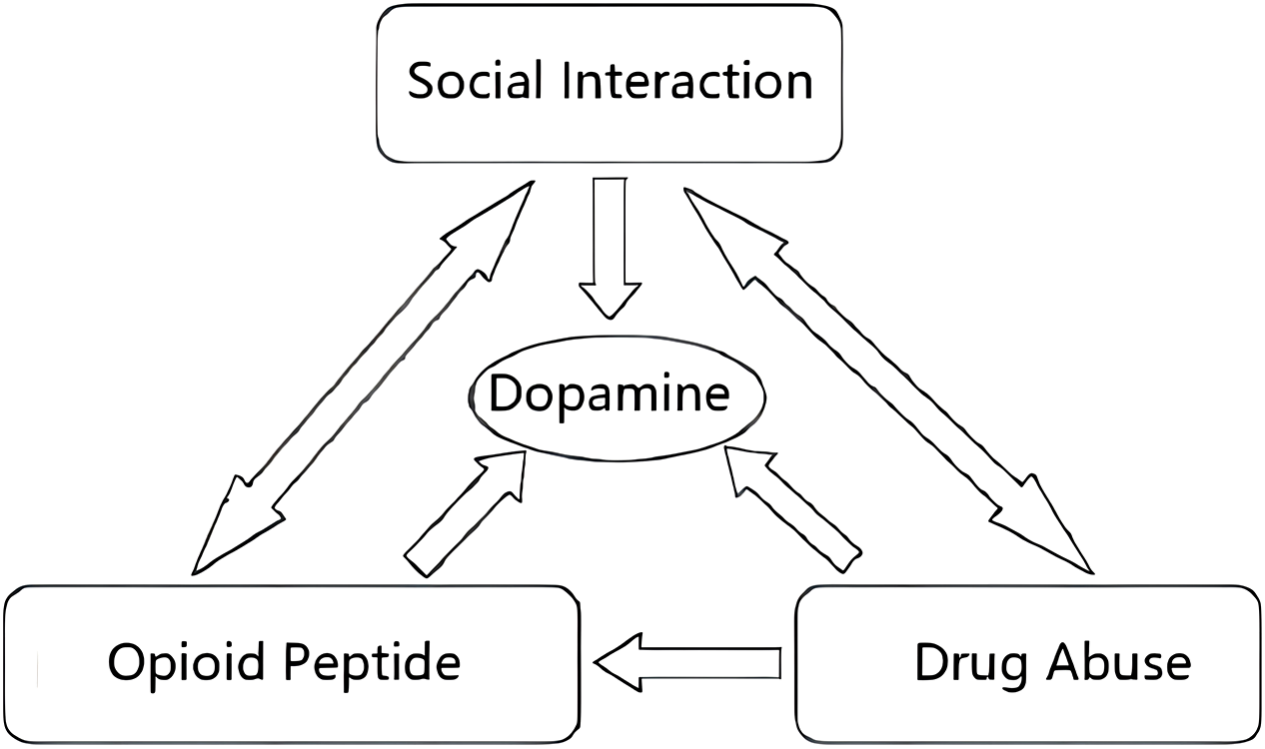
Social interaction and its association with endogenous opioids and drug abuse.

Another critical aspect is community monitoring, which aims to ensure that drug addicts not only abstain from drug use but also adopt healthy lifestyle habits. Additionally, monitoring involves addressing the psychological well-being of drug addicts through regular psychological counseling, medication treatment, and ensuring their psychological stability (44). Consistent psychological and medication treatment plays a significant role in regulating dopamine levels in the brain, thereby influencing the emotions and behavior of an individual. A vital component of community correction is the establishment of a reliable community support system. This entails fostering trustworthy relationships within the community, such as dependable friends and family members, who can offer assistance and support to drug addicts during challenging times or when faced with temptations (45). Furthermore, a comprehensive community support system can use various community resources to help drug addicts effectively cope with life challenges (**Figure 2**).

Correctional efforts should also address psychological deterrence and provide life support for drug addicts. This can be achieved through legal education, the establishment of laws, and the provision of legal support, which can motivate and compel them toward reform. Legal education is significant during the correction phase as it complements and strengthens the legal education provided during the prevention phase. Such education helps drug addicts gain a deeper and more comprehensive understanding of legal provisions and social responsibilities, thereby increasing their legal awareness and promoting law-abiding behavior (46).

Implementing this legal education can potentially reduce the inclination of drug addicts towards criminal behavior by providing them with an understanding of the consequences of their actions and diminishing the dopamine stimulation associated with such behavior. Also, actively advocating for the establishment of laws regarding the criminal behavior of drug addicts from an academic standpoint is crucial. This involves precisely defining the nature of various drug-related crimes and adjusting the severity of legal punishments accordingly. By doing so, drug addicts can receive better correction and education, thereby enabling them to seek dopamine stimulation through legal means rather than resorting to drug use or criminal behavior (47). Furthermore, encouraging the participation of all sectors of society in the correction of drug addicts is essential. By establishing a comprehensive system where the entire society participates in the correction process, comprehensive support can be provided to help individuals overcome addiction and successfully reintegrate into society.

Throughout the entirety of drug addiction treatment, the law assumes a comprehensive and integral role by furnishing regulations, education, and guidance, thereby constituting a complete and cohesive legal framework to bolster the recovery and social reintegration of drug addicts. Additionally, by examining the characteristics and impacts of dopamine, the law can furnish robust legal protection and theoretical framework for the effective treatment of drug addiction.

### 5.4 Guarantee of human rights by correction measures

In the implementation of all corrective measures for drug addicts, safeguarding human rights and respecting personal will are crucial fundamental principles. Human rights are the basic rights inherent to every individual and should be respected and protected regardless of whether there is drug addiction and related criminal behavior.

Firstly, in terms of the community support framework, the establishment of community services, monitoring, and support systems should be based on respecting personal will. For example, when arranging community service tasks, the physical condition and personal abilities of drug addicts should be fully considered to avoid excessive coercion that may lead to resistance. At the same time, during the community monitoring process, measures such as psychological counseling and medication treatment should be carried out with the full knowledge and consent of the addicts, ensuring that they understand the purpose, process, and possible impacts of these measures and respecting their right to choose their own health and lifestyle.

Secondly, the implementation of legal education and legal measures also needs to follow the principle of safeguarding human rights. The content and methods of legal education should be adjusted according to the individual’s acceptance ability and will, avoiding a single, forced indoctrination method. When formulating and implementing relevant laws, the fairness and rationality of the laws should be ensured to avoid discrimination and excessive punishment of drug addicts. For example, when determining the severity of legal punishment, factors such as the criminal motive, personal background, and the addict’s awareness and repentance will of their own behavior should be comprehensively considered, rather than simply relying on the criminal act itself. At the same time, the legal rights and interests of addicts during the treatment and correction process, such as the right to privacy and the right to appeal, should be fully guaranteed to ensure that they can be treated fairly within the legal framework and have the opportunity to express their will and demands.

Only on the basis of safeguarding human rights and respecting personal will can the community support framework, legal education, and other corrective measures we implement truly play their positive roles, helping drug addicts to better reform and reintegrate into society and achieve the ultimate goal of correction.

## 6 Limitation

Although this study has achieved certain results in exploring research topic, there are also some limitations. Firstly, the research sample is mainly sourced from specific source, which may have certain geographical and population limitations and cannot fully represent all relevant situations. Secondly, during the data collection process, there may be certain information biases or omissions, which affect the accuracy of the research results. In addition, the choice of research methods may be limited by various factors, such as time and resources, resulting in a certain impact on the universality of the research results. Finally, this study only focuses on the main aspects of the research, and the consideration of other related factors may not be comprehensive enough. Future research can further expand the sample range, adopt more diverse data collection methods, and comprehensively consider more related factors to improve the accuracy and universality of the research.

## 7 Conclusion

Through analysis and comprehension of the role of dopamine within the framework of drug addiction and criminal behavior, it is evident that a close and intricate association exists between these variables and the dopamine system. Dopamine, given its influence over reward mechanisms and affective states, assumes a pivotal position within this nexus. The aberrant release of dopamine profoundly affects individuals with drug addiction and their propensity for engaging in criminal conduct, underscoring the importance of redressing dopamine dysregulation to reinstate normative functioning. However, additional research endeavors are required to comprehensively apprehend the mechanisms involved in this process.

Future research should be dedicated to the following specific directions: Firstly, on the basis of existing research, further in-depth exploration of the association between drug addiction and dopamine is needed to clarify the specific functional roles of dopamine in this context. This may include the study of differences in the role of dopamine in different types of drug addiction, as well as the interaction mechanism between dopamine and other neurotransmitters in the process of addiction. Secondly, more research should be carried out on interventions capable of modulating and restoring the dopamine system. This not only involves the exploration of drug treatments but also the study of the impact of non-drug intervention methods such as psychotherapy and behavioral therapy on the dopamine system. In addition, given the complexity of drug addiction and criminal behavior, promoting interdisciplinary research is of utmost importance. Integrating the knowledge and methods of multiple fields such as neurobiology, psychology, social sciences, and law will help identify more effective treatment and prevention strategies. For example, studying how social environmental factors affect criminal behavior through influencing the dopamine system, and how legal policies can be combined with the research results of neuroscience and psychology to formulate more targeted intervention measures. Through in-depth research in these directions, we are expected to further optimize the effectiveness of corrective intervention measures and provide a more solid theoretical foundation for understanding the relationship between drug addiction, criminal behavior, and dopamine.

## References

[1] Baler RD Volkow ND. Drug addiction: the neurobiology of disrupted self-control. Trends Mol Med. (2006) 12:559–66. doi: 10.1016/j.molmed.2006.10.005 17070107

[2] Ricci V Ceci F Di Carlo F Lalli A Ciavoni L Mosca A. Cannabis use disorder and dissociation: A report from a prospective first-episode psychosis study. Drug Alcohol Depend. (2021) 229:109118. doi: 10.1016/j.drugalcdep.2021.109118 34688166

[3] Ricci V Ceci F Di Carlo F Di Muzio I Ciavoni L Santangelo M. First episode psychosis with and without the use of cannabis and synthetic cannabinoids: Psychopathology, global functioning and suicidal ideation. Psychiatry Res. (2023) 320:115053. doi: 10.1016/j.psychres.2023.115053 36682093

[4] Belin D Everitt BJ. Cocaine seeking habits depend upon dopamine-dependent serial connectivity linking the ventral with the dorsal striatum. Neuron. (2008) 57:432–41. doi: 10.1016/j.neuron.2007.12.019 18255035

[5] Chiappini S Vaccaro G Mosca A Miuli A Stigliano G Stefanelli G. New trends of drug abuse in custodial settings: A systematic review on the misuse of over-the-counter drugs, prescription-only-medications, and new psychoactive substances. Neurosci Biobehav Rev. (2024) 162:105691. doi: 10.1016/j.neubiorev.2024.105691 38733894

[6] Wise RA Robble MA. Dopamine and addiction. Annu Rev Psychol. (2020) 71:79–106. doi: 10.1146/annurev-psych-010418-103337 31905114

[7] Wise RA. Dopamine, learning and motivation. Nat Rev Neurosci. (2004) 5:483–94. doi: 10.1038/nrn1406 15152198

[8] Ernst M Luciana M. Neuroimaging of the dopamine/reward system in adolescent drug use. CNS spectrums. (2015) 20:427–41. doi: 10.1017/S1092852915000395 PMC4560964 26095977

[9] Koob GF Le Moal M. Addiction and the brain antireward system. Annu Rev Psychol. (2008) 59:29–53. doi: 10.1146/annurev.psych.59.103006.093548 18154498

[10] Robinson TE Berridge KC. The incentive sensitization theory of addiction: some current issues. Philos Trans R Soc B: Biol Sci. (2008) 363:3137–46. doi: 10.1098/rstb.2008.0093 PMC2607325 18640920

[11] Volkow ND Koob GF McLellan AT. Neurobiologic advances from the brain disease model of addiction. New Engl J Med. (2016) 374:363–71. doi: 10.1056/NEJMra1511480 PMC6135257 26816013

[12] Schultz W. Behavioral dopamine signals. Trends Neurosci. (2007) 30:203–10. doi: 10.1016/j.tins.2007.03.007 17400301

[13] Koob GF Le Moal M. Plasticity of reward neurocircuitry and the'dark side'of drug addiction. Nat Neurosci. (2005) 8:1442–4. doi: 10.1038/nn1105-1442 16251985

[14] Belujon P Grace AA. Dopamine system dysregulation in major depressive disorders. Int J Neuropsychopharmacol. (2017) 20:1036–46. doi: 10.1093/ijnp/pyx056 PMC5716179 29106542

[15] Volkow ND Wang GJ Telang F Fowler JS Logan J Childress AR. Cocaine cues and dopamine in dorsal striatum: mechanism of craving in cocaine addiction. J Neurosci. (2006) 26:6583–8. doi: 10.1523/JNEUROSCI.1544-06.2006 PMC6674019 16775146

[16] Cantor D Land KC. Unemployment and crime rates in the post-World War II United States: A theoretical and empirical analysis. Am sociological Rev. (1985) 50(3):317–32. doi: 10.2307/2095542

[17] Moffitt TE Caspi A Harrington H Milne BJ. Males on the life-course-persistent and adolescence-limited antisocial pathways: Follow-up at age 26 years. Dev Psychopathol. (2002) 14:179–207. doi: 10.1017/S0954579402001104 11893092

[18] Sampson RJ Laub JH. Crime in the making: Pathways and turning points through life. Crime Delinquency. (1993) 39:396–6. doi: 10.1177/0011128793039003010

[19] Goldstein RZ Volkow ND. Drug addiction and its underlying neurobiological basis: neuroimaging evidence for the involvement of the frontal cortex. Am J Psychiatry. (2002) 159:1642–52. doi: 10.1176/appi.ajp.159.10.1642 PMC1201373 12359667

[20] Yoder KJ Porges EC Decety J. Amygdala subnuclei connectivity in response to violence reveals unique influences of individual differences in psychopathic traits in a nonforensic sample. Hum Brain Mapp. (2015) 36:1417–28. doi: 10.1002/hbm.22712 PMC4837469 25557777

[21] Mazar N Amir O Ariely D. The dishonesty of honest people: A theory of self-concept maintenance. J marketing Res. (2008) 45:633–44. doi: 10.1509/jmkr.45.6.633

[22] Schultz W. Neuronal reward and decision signals: from theories to data. Physiol Rev. (2015) 95:853–951. doi: 10.1152/physrev.00023.2014 26109341 PMC4491543

[23] Volkow ND Wang GJ Fowler JS Tomasi D Telang F. Addiction: beyond dopamine reward circuitry. Proc Natl Acad Sci. (2011) 108:15037–42. doi: 10.1073/pnas.1010654108 PMC3174598 21402948

[24] Van den Bos W McClure SM. Towards a general model of temporal discounting. J Exp Anal Behav. (2013) 99:58–73. doi: 10.1002/jeab.v99.1 23344988 PMC8127626

[25] Ahmed SH. Validation crisis in animal models of drug addiction: beyond non-disordered drug use toward drug addiction. Neurosci Biobehav Rev. (2010) 35:172–84. doi: 10.1016/j.neubiorev.2010.04.005 20417231

[26] Bechara A. Decision making, impulse control and loss of willpower to resist drugs: a neurocognitive perspective. Nat Neurosci. (2005) 8:1458–63. doi: 10.1038/nn1584 16251988

[27] Naqvi NH Bechara A. The insula and drug addiction: an interoceptive view of pleasure, urges, and decision-making. Brain Structure Funct. (2010) 214:435–50. doi: 10.1007/s00429-010-0268-7 PMC3698865 20512364

[28] Botvin GJ Griffin KW. School-based programmes to prevent alcohol, tobacco and other drug use. Int Rev Psychiatry. (2007) 19:607–15. doi: 10.1080/09540260701797753 18092239

[29] Sansfaçon D Welsh B Waller I. Crime prevention digest II: Comparative analysis of successful community safety. Montreal: Int Centre Prev Crime. (1999) 179014.

[30] Hughes CE Stevens A. What can we learn from the Portuguese decriminalization of illicit drugs? Br J Criminology. (2010) 50:999–1022. doi: 10.1093/bjc/azq038

[31] Ke YT Zhou WH. Progress in research on the neurobiological mechanisms of exercise intervention in drug dependence. Chin J Pharmacol Toxicol. (2015) 29:599–606. doi: 10.3867/j.issn.1000-3002.2015.04.011

[32] Smith MA Pitts EG. Wheel running decreases the positive reinforcing effects of heroin. Pharmacol Rep. (2012) 64:960–4. doi: 10.1016/S1734-1140(12)70891-5 PMC3760409 23087148

[33] Peretti-Watel P Guagliardo V Verger P Pruvost J Mignon P Obadia Y. Sporting activity and drug use: Alcohol, cigarette and cannabis use among elite student athletes. Addiction. (2003) 98:1249–56. doi: 10.1046/j.1360-0443.2003.00490.x 12930212

[34] Correia CJ Benson TA Carey KB. Decreased substance use following increases in alternative behaviors: A preliminary investigation. Addictive Behav. (2005) 30:19–27. doi: 10.1016/j.addbeh.2004.04.006 15561446

[35] Roessler KK. Exercise treatment for drug abuse-A Danish pilot study. Scandinavian J Public Health. (2010) 38:664–9. doi: 10.1177/1403494810371249 20529968

[36] Feng JP Yan Y Lu YL Xu JF Sun B Feng LS. Progress in research on exercise-based drug rehabilitation. China Sport Sci. (2019) 55:3–11. doi: 10.16470/j.csst.2019626

[37] De Leon G Unterrainer HF. The therapeutic community: A unique social psychological approach to the treatment of addictions and related disorders. Front Psychiatry. (2020) 11:786. doi: 10.3389/fpsyt.2020.00786 32848950 PMC7424041

[38] Dutra L Stathopoulou G Basden SL Leyro TM Powers MB Otto MW. A meta-analytic review of psychosocial interventions for substance use disorders. Am J Psychiatry. (2008) 165:179–87. doi: 10.1176/appi.ajp.2007.06111851 18198270

[39] United Nations Office on Drugs and Crime. From Coercion to Cohesion: Treating Drug Dependence through Health Care, not Punishment. (2010).

[40] McSweeney T Stevens A Hunt N Turnbull PJ. Twisting arms or a helping hand? Assessing the impact of ‘coerced’and comparable ‘voluntary’drug treatment options. Br J Criminology. (2007) 47:470–90. doi: 10.1093/bjc/azl087

[41] Maruna S. Making good (Vol. 86). Washington, DC: American Psychological Association (2001).

[42] Ashford RD Brown AM Ryding R Curtis B. Building recovery ready communities: The recovery ready ecosystem model and community framework. Addict Res Theory. (2020) 28:1–11. doi: 10.1080/16066359.2019.1571191

[43] Perry AE Neilson M Martyn-St James M Glanville JM Woodhouse R Godfrey C. Interventions for drug-using offenders with co-occurring mental illness. Cochrane Database Systematic Rev. (2015) 6. doi: 10.1002/14651858.CD010862.pub2 26034938

[44] Nawi AM Ismail R Ibrahim F Hassan MR Manaf MRA Amit N. Risk and protective factors of drug abuse among adolescents: a systematic review. BMC Public Health. (2021) 21:1–15. doi: 10.1186/s12889-021-11906-2 34774013 PMC8590764

[45] Mennis J Stahler GJ Mason MJ. Risky substance use environments and addiction: a new frontier for environmental justice research. Int J Environ Res Public Health. (2016) 13:607. doi: 10.3390/ijerph13060607 27322303 PMC4924064

[46] Bonta J Andrews DA. Risk-need-responsivity model for offender assessment and rehabilitation. Rehabilitation. (2007) 6:1–22.

[47] Caulkins JP Reuter P. How drug enforcement affects drug prices. Crime Justice. (2010) 39:213–71. doi: 10.1086/652386

